# Can vigorous physical activity mitigate the effect of systemic inflammation on cognitive performance? Results from a large older community dwelling population in The Netherlands

**DOI:** 10.1177/13872877251386480

**Published:** 2025-10-16

**Authors:** Anne Fink, Constantin Reinke, Benjamin Aretz, Michael T Heneka, Gabriele Doblhammer

**Affiliations:** 1 German Center for Neurodegenerative Diseases, Bonn, Germany; 2 Department of Child and Adolescent Psychiatry, Rostock University Medical Center, Rostock, Germany; 3 Institute of Sociology and Demography, University of Rostock, Rostock, Germany; 4 Institute for Medical Biometry, Informatics and Epidemiology, University Hospital Bonn, University of Bonn, Bonn, Germany; 5 Luxembourg Centre for Systems Biomedicine, University of Luxembourg, Esch-Belval, Luxembourg

**Keywords:** Alzheimer's disease, cognitive performance, cohort study, physical activity, systemic inflammation

## Abstract

**Background:**

Elevated systemic inflammation is associated with poorer cognitive function, while vigorous physical activity enhances cognition.

**Objective:**

This study examines whether physical activity moderates the relationship between systemic inflammation and cognitive performance.

**Methods:**

We analyzed 24,661 adults (50+) from the Dutch Lifelines cohort across two waves. Cognitive performance was assessed via a validated composite score from the Cogstate Brief Battery, with higher scores indicating lower cognitive performance. Leukocyte count (3–11 × 10^9^ cells/liter) served as a systemic inflammation biomarker, categorized as low (<6.5 × 10^9^ cells/liter) or high (≥6.5 × 10^9^ cells/liter). We used the Short Questionnaire to Assess Health-Enhancing Physical Activity to define moderate (0, 1–149, 150+ minutes) and vigorous (0, 1–74, 75+ minutes) physical activity. We performed linear regression models to examine the effect of inflammation and vigorous physical activity on cognition, adjusting for moderate physical activity and covariates. An interaction effect analyzed the potential moderation of vigorous physical activity.

**Results:**

Individuals with high systemic inflammation (SI) levels in both waves exhibited significantly longer reaction times (b = 0.062 [95% confidence interval: 0.002; 0.122]) compared to those with low SI levels in both waves. Individuals who engaged in 1–74 min or 75+ minutes of vigorous physical activity had significantly faster reaction times (1–74: b = −0.114 [−0.193; −0.034], 75+: b = −0.160 [−0.210; −0.111]) than those who did not. The interaction term was insignificant.

**Conclusions:**

Although vigorous physical activity is protective, it cannot mitigate the negative association between systemic inflammation and cognition. Nevertheless, promoting vigorous physical activity in an ageing population can be an effective strategy for preventing or delaying cognitive decline.

## Introduction

The increasing prevalence of dementia, both globally and particularly in Europe, poses a significant challenge to public health. As populations age, the number of individuals affected by this condition is expected to rise significantly from almost 50 million in 2015 to over 152 million in 2050 worldwide.^1^ While the urgent need for effective strategies against cognitive impairment and the development of dementia is widely acknowledged, there is still a lack of understanding of the potential effectiveness of primary prevention interventions in the older population.^2^

Newer studies revealed that systemic inflammation processes are one of the most important risk factors for neurodegeneration.^3^ Several studies have shown that chronic systemic inflammation is associated with cognitive decline, affecting essential areas like memory, attention, and processing speed, as well as an increased risk of dementia.^4–8^ One hypothesis is that inflammatory response causes the disruption of neuronal function and subsequent changes in brain structure.^9^ Although systemic inflammation may be the response to the pathogenesis of cognitive decline or dementia, systemic inflammation may still influence the progression and clinical manifestation of the disease.^10^

An elevated white blood cell count may serve as an early indicator of systemic inflammation and is associated with the progression of various diseases. A slightly elevated white blood cell count, even within the normal range, could indicate a higher likelihood of increased systemic inflammation and subclinical disease. In addition to an increased risk of developing cancer, cardiovascular diseases, type 2 diabetes, and other age-related diseases, as well as an increased risk of all-cause mortality^11^ an elevated white blood cell count is also associated with reduced cognitive performance.^12^

In contrast, physical activity is commonly linked to better cognitive performance. Regular exercise has been shown to improve several cognitive functions, including processing speed, memory, and executive function.^13^ It is believed that the advantageous outcomes of physical activity on cognition are mediated through various mechanisms. These include promoting neuroplasticity, increasing brain-derived neurotrophic factor levels, improving cerebral blood flow, as well as reducing systemic chronic inflammation by reducing adipose tissue, enhancing the release of anti-inflammatory myokines, improving endothelial function, and modulating metabolic health.^14–16^ Physical activity is therefore an important modifiable risk factor for cognitive decline and dementia in the absence of curative treatment options.^17^

While it is clear that both systemic inflammation and physical activity independently affect cognitive performance, their interactions are less well understood. This large cohort study uses an older community-dwelling setting in the Netherlands to explore whether vigorous physical activity can mitigate the effect of systemic inflammation on cognitive performance. Our hypothesis is that vigorous physical activity moderates the relationship between leukocyte count and cognitive performance by altering the strength of the relationship. We therefore theorize that elevated leukocyte counts affect vigorously active and inactive individuals differently.

## Methods

### Data

The current study uses baseline and follow-up data (2006–2017) from the Lifelines Cohort Study and Biobank. Information on the study design and objectives of Lifelines has been previously described elsewhere.^18,19^ In summary, Lifelines is a multi-disciplinary prospective population-based cohort study examining in a unique three-generation design the health and health-related behaviors of 167,729 persons living in the North of the Netherlands. It employs a broad range of investigative procedures in assessing the biomedical, socio-demographic, behavioral, physical and psychological factors which contribute to the health and disease of the general population, with a special focus on multi-morbidity and complex genetics. Lifelines was conducted in accordance with the guidelines of the Declaration of Helsinki. All procedures involving human subjects were approved by the Medical Ethics Committee of the University Medical Center Groningen (UMCG), and written informed consent was obtained from all participants. Our study included adults aged 50 years and above (N = 24,661), who had valid information on body height, body weight, systolic blood pressure, and physical activity at baseline (2006–2013), leukocyte count measurements at baseline (2006–2013) and follow-up (2014–2017), and cognitive performance measurements at follow-up (2014–2017).

### Outcome: cognitive performance

The CogState Brief Battery (CBB) is a computer-administered cognitive test battery that takes about 10 min to administer. It consists of four cognitive tasks that measure cognitive functions: Psychomotor function, attention, working memory, and memory.^20^ The CBB has shown its effectiveness and sensitivity in detecting cognitive impairment in conditions like Alzheimer's disease (AD) and mild cognitive impairment, and in assessing cognitive changes in the preclinical stages of AD.^20^ This makes it a valuable tool in the field of cognitive neuroscience. Following two previous studies,^21,22^ we used two cognitive processing speed measures from the CBB. First, the Detection Task, which measures psychomotor function – the coordination of sensory or cognitive processes and motor activity. Second, the Identification Task, which measures attention. For both tasks, the primary outcome was reaction time in milliseconds (speed). A base 10 logarithmic transformation (log10) was used to normalize the distribution. The log-transformed values were z-standardized for 5-year age groups starting at age 50 and summed up to a composite score measuring cognitive performance. The resulting score, measured as the combined z-standardized and log-transformed reaction times from the Detection and Identification tasks of CBB, ranged from −8.21 to 13.24, with higher reaction times indicating poorer cognitive function. Detection Task and Identification Task were shown to have excellent specificity, validity and reliability.^23^

### Exposure: marker of systemic inflammation (SI): leukocyte count

As a proxy biomarker of systemic inflammation (SI), we used leukocyte count within the normal range of 3 to 11 × 10^9^ cells per liter in EDTA-blood samples at baseline (2006–2013) and follow-up (2014–2017). We excluded all individuals who had a leukocyte count of less than 3 or greater than 11 at either the baseline or at follow-up to reduce the possibility that conditions such as acute infections or leukemia, could bias the findings due to undetected and uncontrolled inflammation-related diseases and thus the computerized tests of the CBB.^10^ The highest 25% of the leukocyte count (>=6.5 × 10^9^ cells per liter) was defined as high SI levels.^24,25^ Values below this cutoff were defined as low SI levels. We performed four groups and distinguished between (1) individuals who had low SI at baseline and at follow-up (low-low), (2) individuals who had elevated SI at baseline but low SI at follow-up (high-low), (3) individuals who had low SI at baseline but elevated SI at follow-up (low-high), and (4) individuals who had elevated SI at baseline and at follow-up (high-high).

### Moderator variable: vigorous physical activity

Physical activity was assessed using the short questionnaire to assess health-enhancing physical activity (SQUASH), a questionnaire to measure the habitual physical activity level of adults, developed by the Dutch National Institute of Public Health and the Environment. The questionnaire consists of questions on four categories of activities: commuting (walking or bicycling to work), leisure time (walking, bicycling, gardening, odd jobs, sports), household (light such as cooking, washing dishes, ironing, child care, or intense such as scrubbing floor, walking with heavy shopping bags), and activities at work (light such as sitting/standing with some walking, e.g. a desk job, or intense such as regularly lifting heavy objects at work). For each activity, the frequency (number of days), duration (in hours and minutes), and intensity (effort: slow/moderate/fast) are recorded for a normal week.^26^ Examples of activities were given to make it easier for participants to assess the intensity of the activities. The information on intensity was converted to metabolic equivalent of task (MET) scores. Moderate physical activity was defined as having a (MET score of 4–6.5 for persons aged 50–55, and 3–5 for persons over 55. Vigorous physical activity was defined as having a MET score greater than 6.5 for persons aged 18 to 55 years, and greater than 5 METs for persons older than 55 years.^27^ Detailed information on SQUASH can be found here.^28^ We had information on the number of minutes per week in each activity category and based our analysis on the WHO guidelines^29^ on physical activity . Therefore, we differentiated between 0 min, less than 75 min, and 75 min or more for vigorous activities. Since high-intensity exercise has been shown to have the greatest impact on the inflammatory profile, we focus on vigorous physical activity.^16^

### Covariates

The following covariates were measured at baseline: sex (male, female), age, education (low, medium, high, missing), level of moderate physical activity (0 min, less than 150 min, 150 min or more), smoking status (nonsmoker, current smoker, missing), and self-reported diagnoses of diabetes, depression, stroke, myocardial infarction, heart failure, or Parkinson's disease. Additionally, we included obesity (body mass index ≥30 kg/m² derived from measured body height and weight during assessment) and systolic blood pressure measured during assessment. We used the speed measures recorded by the CBB as our primary outcome. This implies that participants who responded quickly to the tasks may still have provided inaccurate responses. To account for this possible response accuracy, we calculated a composite score of response accuracy measured by the number of correct responses divided by the number of total responses in the two CBB domains.

### Statistical analyses

We used one-way analysis of variance and Bonferroni-corrected post hoc test (for covariates with more than two categories) to compare cognitive function scores between groups of SI levels, vigorous physical activity, and covariates including moderate physical activity levels. Statistical significance was set at p < 0.05. With correction for multiple comparisons**,** the adjusted alpha level was calculated by dividing the standard threshold (0.05) by the number of comparisons conducted. Accordingly, a result was deemed significant only if the p-value was below the corrected threshold.

To estimate cognitive performance, we used a linear regression model with robust standard errors (Breusch-Pagan with p < 0.001) as a function of SI levels, vigorous and moderate physical activity, sex, age, education, smoking status, obesity, and self-reported diagnoses. A second model additionally included an interaction effect between vigorous physical activity and SI level groups. To account for selection bias, we used inverse probability weighting. Stabilized weights were obtained from the quotient of a logistic response model as a function of all included variables of the association model and a logistic response model with age, sex, and education only.^30,31^

## Results

The study population consisted of 24,661 individuals aged 50 to 88 years. Mean age was 58.2 years with a standard deviation of 6.6 years. Mean follow-up time between the two waves was 44.3 months (95% confidence interval [44.1; 44.3]; minimum: 11 months, maximum: 122 months). The cognitive performance score had a mean value of −0.019 [−0.040; 0.003], and Supplemental Figure 1 displays its distribution. Higher values correspond to poorer cognitive performance. Table 1 presents the characteristics of the study population and the associations between the dependent and independent variables. Individuals with low levels of inflammation in both waves (low-low) had an average cognitive function score of −0.051 [−0.078; −0.024]. In contrast, those with high levels of inflammation in both waves (high-high) had an average cognitive function score of 0.080 [0.027; 0.134], indicating significantly poorer cognitive performance (p < 0.01). Individuals who were vigorously active for 1 to 74 min per week (−0.155 [−0.232; −0.078], p < 0.001) or for 75 min or more per week (−0.232 [−0.274; −0.191], p < 0.001) had a significantly better cognitive function than those who were vigorously inactive (0.071 [0.045; 0.098]). Regarding moderate physical activity the best cognitive performance was observed among individuals with 1 to 149 min per week (−0.118 [−0.160; −0.076]). Those with 0 min per week had a score of 0.107 [−0.028; −0.062], and those with at least 150 min had a score of 0.011 [−0.019; 0.041], p < 0.001) indicating worse cognitive performance. Men had a better average cognitive function (−0.094 [−0.127; −0.062]) than women (0.042 [0.013; 0.071]). Furthermore, there was an education gradient observed, as individuals with lower education exhibited significantly poorer cognitive function (0.307 [0.270; 0.343]) compared to those with medium (−0.037 [−0.076; 0.001], p < 0.001) or high (−0.452 [−0.486; −0.418], p < 0.001) education. Individuals who reported having diabetes, stroke, or heart failure had significantly poorer cognitive function compared to those without these diseases. We found a negative gradient between systolic blood pressure and cognitive performance. Specifically, individuals with a systolic blood pressure of 140 mmHg and over had an average score of 0.080 [0.037; 0.124], while those with a systolic blood pressure lower than 120 mmHg had an average score of −0.091 [−0.132; −0.051] (p < 0.01). Additionally, we found that individuals without obesity had better cognitive function (−0.037 [−0.061; −0.014]) compared to individuals with obesity (0.079 [0.025; 0.133]).

**Table 1.** Characteristics of study participants and associations of predictor variables with mean of score of cognitive performance. Lower cognitive function scores reflect better cognitive performance.

Variable	Categories	N	%	Mean of cognitive function	95% Confidence interval		p
Levels of systemic inflammation in wave 1 and 2	Low-low	15,780	64.0	−0.051	−0.078	−0.024	**<0** **.** **001**
	High-low	1977	8.0	−0.047	−0.122	0.028	
	Low-high	2756	11.2	0.036	−0.030	0.102	
	High-high	4148	16.8	0.080	0.027	0.134	
Vigorous physical activity	0 min/week	16,888	68.5	0.071	0.045	0.098	**<0**.**001**
	1–74 min/week	1801	7.3	−0.155	−0.232	−0.078	
	>=75 min/week	5972	24.2	−0.232	−0.274	−0.191	
Moderate physical activity	0 min/week	5758	23.4	0.017	−0.028	0.062	**<0**.**001**
	1–149 min/week	5947	24.1	−0.118	−0.160	−0.076	
	>=150 min/week	12,956	52.5	0.011	−0.019	0.041	
Sex	Male	10,988	44.6	−0.094	−0.127	−0.062	**<0**.**001**
	Female	13,673	55.4	0.042	0.013	0.071	
Age group	50–54	8923	36.2	−0.023	−0.060	0.012	0.999
	55–59	5932	24.1	−0.021	−0.065	0.023	
	60–64	5327	21.6	−0.019	−0.057	0.035	
	65–69	3066	12.4	−0.011	−0.071	0.048	
	70–74	1080	4.4	−0.027	−0.128	0.074	
	75–79	273	1.1	−0.012	−0.213	0.188	
	80–84	52	0.2	−0.030	−0.489	0.429	
	85+	8	0.03	0.083	−1.293	1.458	
Education	Low	9635	39.1	0.307	0.270	0.343	**<0**.**001**
	Medium	7453	30.2	−0.037	−0.076	0.001	
	High	7062	28.6	−0.452	−0.486	−0.418	
	na	511	2.1	0.102	−0.057	0.261	
Diabetes	No	23,428	95.0	−0.031	−0.053	−0.009	**<0**.**001**
	Yes	1233	5.0	0.221	0.126	0.315	
Depression	No	22,311	90.5	−0.023	−0.046	−0.001	0.210
	Yes	2350	9.5	0.024	−0.047	0.094	
Stroke	No	24,385	98.9	−0.022	−0.044	−0.001	**0**.**002**
	Yes	276	1.1	0.299	0.087	0.512	
Myocardial infarction	No	24,127	97.8	−0.021	−0.043	0.001	0.183
	Yes	534	2.2	0.079	−0.075	0.234	
Heart failure	No	24,379	98.9	−0.021	−0.043	0.000	**0**.**038**
	Yes	282	1.1	0.193	−0.030	0.417	
Parkinson's disease	No	24,642	99.9	−0.019	−0.041	0.002	0.377
	Yes	19	0.1	0.330	−0.367	1.027	
Systolic blood pressure	<120	6768	27.4	−0.091	−0.132	−0.051	**<0**.**001**
	120–129	6223	25.2	−0.053	−0.095	−0.011	
	130–139	5398	21.9	−0.003	−0.050	0.044	
	>=140	6272	25.4	0.080	0.037	0.124	
Obesity	No	20,723	84.0	−0.037	−0.061	−0.014	**<0**.**001**
	Yes	3938	16.0	0.079	0.025	0.133	
Smoking status	No	21,259	86.2	−0.026	−0.058	0.007	0.343
	Yes	3262	13.2	0.019	−0.042	0.079	
	na	140	0.6	0.160	−0.115	0.434	
Total		24,661	100.0	−0.019	−0.040	0.003	

na: not available; min: minutes. Bold type indicates statistically significant differences or effect sizes.

*Source:* Lifelines data wave 1 2006–2013 and wave 2 2014–2017, own calculation.

Individuals with depression, myocardial infarction, or Parkinson's disease (PD) did not differ significantly from those without these diseases. There was no statistically significant difference in average cognitive function between nonsmokers (−0.026 [−0.058; 0.007]) and smokers (0.019 [−0.042; 0.079]).

### Model results

After adjusting for cognitive task accuracy, age, sex, physical activity, education, medical conditions, and smoking status, individuals with high SI levels in both waves exhibited significantly longer reaction times (b = 0.062 [0.002; 0.122]) compared to those with low SI levels in both waves. This suggests that consistently high levels of systemic inflammation are associated with poorer cognitive function (Table 2, model 1). Individuals with a high level of SI at wave one but a low level at wave two and those with a low level of SI at wave one but a high level at wave two did not differ significantly from the reference group. Individuals who engaged in vigorous physical activity for 1 to 74 min per week (b = −0.114 [−0.193; −0.034]) or 75 or more minutes per week (b = −0.160 [−0.210; −0.111]) had significantly faster reaction times compared to those who did not, indicating a positive dose-response association between vigorous physical activity and cognitive function. Significantly faster reaction times were observed for individuals reporting 1 to 149 min per week moderate physical activity compared to moderately inactive persons (b = −0.065 [−0.124;−0.005]). Additionally, women exhibited significantly longer reaction times than men (b = 0.111 [0.067; 0.155]), and those with medium or high education levels had faster reaction times than those with low education (medium: b = −0.246 [−0.299; −0.193], high: b = −0.552 [−0.603; −0.500]). Only two reported comorbidities, diabetes, and stroke, were significantly associated with worse cognitive function. Diabetes had a coefficient of b = 0.131 [0.036; 0.226], while stroke had a coefficient of b = 0.291 [0.082; 0.500]. A significant effect of systolic blood pressure was found, indicating that higher systolic blood pressure is associated with worse cognitive function, as reaction time increased by 0.003 [0.001; 0.004] per unit increase in systolic blood pressure.

**Table 2.** Results of linear regression models to identify impact of systemic inflammation (SI) and vigorous physical activity on cognitive performance including interaction terms in model 2. Lower cognitive function scores reflect better cognitive performance.

		Model 1				Model 2			
Variable	Categories	Coefficient b	p	95% Confidence interval		Coefficient b	p	95% Confidence interval	
Levels of SI in wave 1 and 2	Low-low	0				0			
	High-low	−0.023	0.563	−0.102	0.055	−0.044	0.361	−0.138	0.050
	Low-high	0.055	0.110	−0.012	0.122	0.033	0.429	0.049	0.115
	High-high	0.062	0.044	0.002	0.122	0.056	0.118	−0.014	0.125
Vigorous physical activity	0 min/week	0				0			
	1–74 min/week	−0.114	**0** **.** **005**	−0.193	−0.034	−0.124	**0**.**012**	−0.221	−0.027
	>=75 min/week	−0.160	**<0**.**001**	−0.210	−0.111	−0.176	**<0**.**001**	−0.235	−0.117
Levels of SI × vigorous physical activity	High-low × 1–74 min/week					0.156	0.304	−0.142	0.454
	High-low × >=75 min/week					0.035	0.708	−0.150	0.221
	Low-high × 1–74 min/week					−0.014	0.914	−0.265	0.237
	Low-high × >=75 min/week					0.090	0.258	−0.066	0.247
	High-high × 1–74 min/week					−0.008	0.949	−0.246	0.230
	High-high × >=75 min/week					0.027	0.715	−0.117	0.170
Moderate physical activity	0 min/week	0				0			
	1–149 min/week	−0.065	**0**.**033**	−0.124	−0.005	−0.065	**0**.**032**	−0.125	−0.006
	>=150 min/week	0.012	0.658	−0.040	0.064	0.012	0.658	−0.040	0.064
Sex	Male	0				0			
	Female	0.111	**<0**.**001**	0.067	0.155	0.111	**<0**.**001**	0.067	0.155
Education	Low	0				0			
	Medium	−0.246	**<0**.**001**	−0.299	−0.193	−0.247	**<0**.**001**	−0.300	−0.194
	High	−0.552	**<0**.**001**	−0.603	−0.500	−0.552	**<0**.**001**	−0.603	−0.500
	na	−0.136	0.082	−0.288	0.017	−0.136	0.081	−0.289	0.017
Diabetes	No	0				0			
	Yes	0.131	**0**.**007**	0.036	0.226	0.131	**0**.**007**	0.036	0.226
Depression	No	0				0			
	Yes	0.031	0.398	−0.041	0.102	0.031	0.390	−0.040	0.103
Stroke	No	0				0			
	Yes	0.291	**0**.**006**	0.082	0.500	0.291	**0**.**006**	0.082	0.500
Myocardial infarction	No	0				0			
	Yes	0.044	0.577	−0.110	0.198	0.043	0.582	−0.111	0.198
Heart failure	No	0				0			
	Yes	0.100	0.370	−0.119	0.320	0.101	0.369	−0.119	0.320
Parkinson's disease	No	0				0			
	Yes	0.251	0.438	−0.384	0.886	0.251	0.439	−0.385	0.887
Systolic blood pressure in mmHg		0.003	**<0**.**001**	0.001	0.004	0.003	**<0**.**001**	0.001	0.004
Obesity	No	0				0			
	Yes	−0.034	0.250	−0.093	0.024	−0.034	0.254	−0.092	0.024
Smoking status	No	0				0			
	Yes	−0.020	0.552	−0.086	0.046	−0.020	0.558	−0.086	0.046
	na	0.103	0.442	−0.160	0.366	0.103	0.440	−0.159	0.366
Constant		−0.121	0.352	−0.377	0.134	0.115	0.380	−0.371	0.141
Adjusted R²		0.0868				0.0869			
N		24,661				24,661			

Models are adjusted for age and accuracy of cognitive tasks.

SI: systemic inflammation; na: not available; min: minutes. Bold type indicates statistically significant differences or effect sizes.

*Source:* Lifelines data wave 1 2006–2013 and wave 2 2014–2017, own calculation.

Depression, myocardial infarction, heart failure, PD, obesity and smoking did not show a significant association.

### Interaction model

The analysis including the interaction term of systemic inflammation and vigorous physical activity (model 2) showed that vigorous physical activity was associated with faster reaction times with a coefficient of b = −0.124 [−0.221; −0.027] for 1 to 74 min per week and b = −0.176 [−0.235; −0.117] for 75 or more minutes per week. Individuals with high SI levels in both waves exhibited longer reaction times (b = 0.056 [−0.014; 0.125]) compared to those with low SI levels in both waves, although this was not statistically significant. Individuals with a high level of SI at wave one but a low level at wave two and those with a low level of SI at wave one but a high level at wave two did not differ significantly from the reference group. The interaction term itself did not show significant estimates, meaning that vigorous physical activity did not moderate the relationship between leukocyte count and cognitive performance.

## Discussion

Our study examined the potential moderator effect of vigorous physical activity on the relationship between the systemic inflammation levels within the normal range and cognition in individuals over two waves in an older community-dwelling population. In line with previous studies,^4–6,12^ we found that elevated levels of systemic inflammation are associated with poorer cognitive performance. Individuals with consistently high inflammation levels in both waves (high-high) had significantly worse cognitive function compared to those with consistently low levels of inflammation (low-low). This finding supports the hypothesis that chronic inflammation can have detrimental effects on cognitive health. Amongst other factors, chronic systemic inflammation can be promoted by a number of risk factors, including chronic infections, autoimmune diseases, an inflammatory diet (high in fat and sodium), physical inactivity, visceral obesity, microbiome dysbiosis, smoking, poor sleep, social isolation, chronic stress, and exposure to toxic substances such as air pollutants, pesticides, or industrial chemicals.^10,32^ Also, the ageing process itself may lead to elevated chronic systemic inflammation, which is referred to as immunosenescence.^10^ Mechanistically, inflammatory cytokines may disrupt neuronal function and alter brain structure, impacting cognitive processes such as synaptic plasticity and neurotransmitter metabolism, or even drive neurodegenerative processes.^3,9^ Our results underscore the importance of monitoring, preventing, or possibly treating systemic inflammation as it represents a potential risk factor for cognitive decline and age-related diseases.

Further, we were able to confirm the positive impact of vigorous physical activity on cognitive performance. Vigorous physical activity was associated with faster reaction times, indicating better cognitive performance, even after adjusting for sociodemographics, lifestyle factors, moderate physical activity levels and important comorbidities associated with cognitive performance. This aligns with the existing literature suggesting that vigorous physical activity promotes cognitive performance.^13,33^ A study of a small number of institutionalized and cognitively impaired older women also showed that vigorous physical activity led to a reduction in inflammation and an improvement in global cognition within 28 weeks.^34^ Interestingly, we found a reduced cognitive performance with increasing number of minutes with moderate physical activity. This may be due to measurement errors and biases caused by misreporting, such as social desirability bias, or by difficulty remembering and estimating the frequency and duration of different activities. This can lead to an overestimation of moderate activities..^35,36^ Even though the interaction effects between moderate and vigorous physical activity were not significant in the sensitivity analysis, it is possible that some of the individuals who engaged in 1–149 min of moderate activity also engaged in vigorous activity, which could have led to better cognitive scores. Regarding our hypothesis, our interaction analysis revealed no significant interaction terms, which means that vigorous physical activity did not moderate the association between inflammation levels and cognitive performance. However, vigorous physical activity was associated with positive cognitive performance while controlling for systemic inflammation levels. This suggests that vigorous physical activity is a universal protective factor that promotes cognitive health and emerges as a promising modifiable factor for mitigating cognitive decline. However, our results do not show that the absence of vigorous physical activity is the mechanism by which systemic inflammation is associated with poor cognitive performance.

Our study has several strengths and limitations. One strength is the use of a large, population-based cohort with repeated measurements of leukocyte count. This allowed us to examine the temporal relationship between inflammation levels and cognition, and to control for potential confounding factors. Another strength is the use of a validated composite score from the CBB that assessed the two domains attention and psychomotor function to measure cognitive performance, which has been shown to be more sensitive to both AD-related cognitive impairment and decline than scores from the individual CBB tasks.^20,23^

A limitation of our study is the reliance on self-reported physical activity, which may be subject to recall bias and social desirability. In addition, we used leukocyte count as a proxy for systemic inflammation, which may not capture the complexity and heterogeneity of the inflammatory response. Future studies may benefit from using more specific biomarkers of inflammation, such as cytokines, chemokines, and complement factors. Furthermore, we only have information on the leukocyte count at the two-time points of the two waves. Therefore, we cannot rule out that critical events, such as severe acute infections or surgery,^10^ may have occurred in the meantime, which could influence cognitive performance at the time of the second wave. To measure physical activity, we distinguished between people who are vigorously physically active and those who are not. The “inactive” group is therefore quite heterogeneous and consists of people who are not active at all and people who are only moderately active. But, we controlled for moderate physical activity levels. Further, it is difficult to distinguish minutes of moderate and vigorous physical activity accurately from a brief self-report questionnaire. Another limitation is the relatively short time interval of a few years between the two waves, so that we cannot be sure that people had already reduced their physical activity level at the beginning of the study due to the onset of cognitive problems. We only have information on cognitive performance at the time of the second wave. The attrition rate between the first and second wave was significant, with almost 30% of respondents not participating in the second wave.^18^ To address the issue of a higher drop-out probability for individuals with worse cognitive function at baseline, we applied inverse probability weighting. We lacked information on environmental factors that may also impact inflammation levels, cognitive performance, and the relationship between the two. Finally, our analyses were constrained to adjust for self-reported comorbidities, obesity, and smoking status.

The implications of our study are relevant for public health and clinical practice. The results of our study indicate that elevated systemic inflammation is a risk factor for cognitive impairment in older adults whereas vigorous physical activity is a protective factor. Therefore, promoting vigorous physical activity among the ageing population may be an effective strategy to prevent or delay cognitive decline and dementia. Moreover, screening for systemic inflammation and monitoring cognitive function may help identify individuals who are at high risk of cognitive impairment and who may benefit from early intervention.

### Conclusions

In conclusion, the results of this study highlight the complex interplay between systemic inflammation, vigorous physical activity, and cognitive performance. Vigorous physical activity is associated with positive cognitive performance when controlling for systemic inflammation. Simultaneously, the findings underscore the importance of controlling inflammation markers and cardiovascular risk factors to prevent cognitive impairments. Future research should continue to explore these relationships to develop targeted interventions for promoting cognitive health in the population.

## Supplemental Material

sj-docx-1-alz-10.1177_13872877251386480 - Supplemental material for Can vigorous physical activity mitigate the effect of systemic inflammation on cognitive performance? Results from a large older community dwelling population in The Netherlands Supplemental material, sj-docx-1-alz-10.1177_13872877251386480 for Can vigorous physical activity mitigate the effect of systemic inflammation on cognitive performance? Results from a large older community dwelling population in The Netherlands by Anne Fink, Constantin Reinke, Benjamin Aretz, Michael T Heneka and Gabriele Doblhammer in Journal of Alzheimer's Disease
