# Supplementary material for: Can vigorous physical activity mitigate the effect of systemic inflammation on cognitive performance? Results from a large older community dwelling population in The Netherlands
Source: J Alzheimers Dis. 2025 Oct 16;108(3):1369–77. doi: 10.1177/13872877251386480 (PMC12647380; doi:10.1177/13872877251386480)

**Supplemental Material**

**Can vigorous physical activity mitigate the effect of systemic inflammation on cognitive performance? Results from a large older community dwelling population in the Netherlands**

**Supplemental Figure 1.** Distribution of cognitive performance score. Lower cognitive function scores reflect better cognitive performance. Source: Lifelines data wave 2 2014-2017, own calculation.


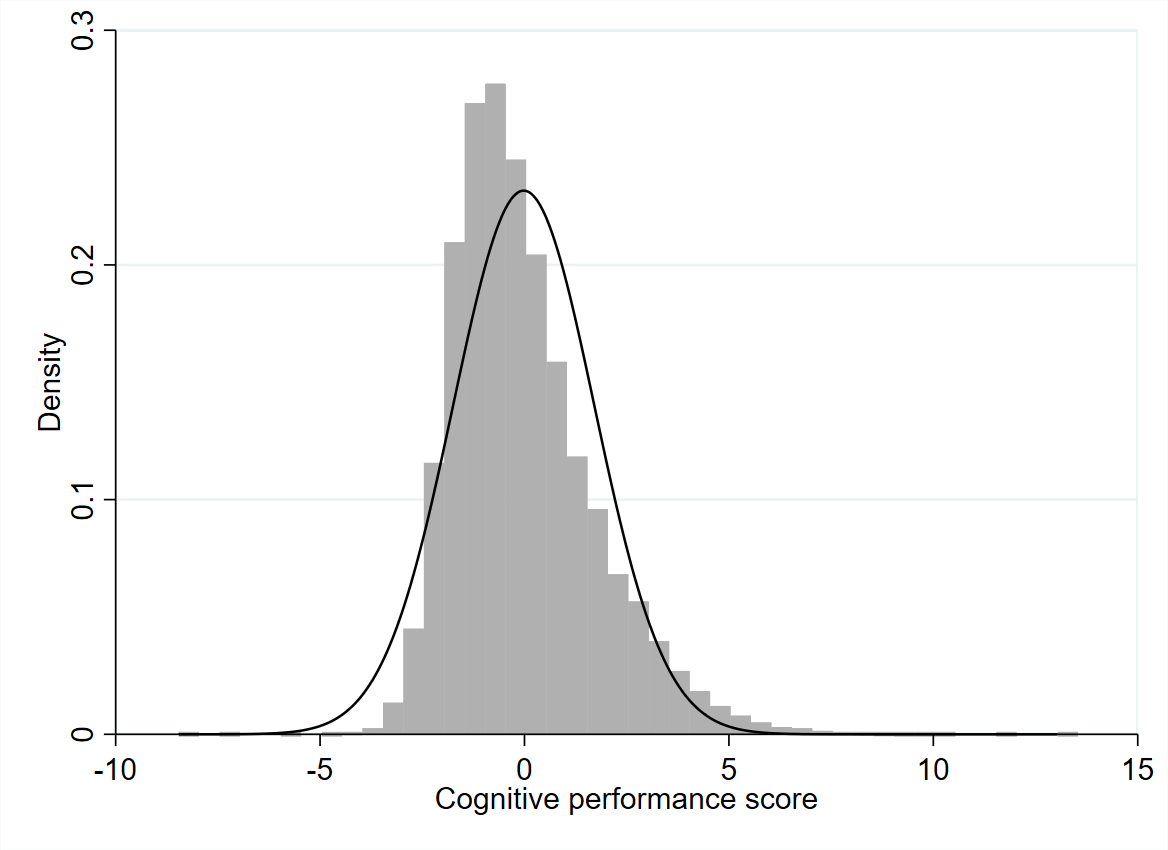

Supplement: sj-docx-1-alz-10.1177_13872877251386480 - Supplemental material for Can vigorous physical activity mitigate the effect of systemic inflammation on cognitive performance? Results from a large older community dwelling population in The Netherlands [file sj-docx-1-alz-10.1177_13872877251386480.docx]
